# P-188. Intrafamilial Aggregation of Adult T-Cell Leukemia Among HTLV-1 Carriers in Peru

**DOI:** 10.1093/ofid/ofaf695.411

**Published:** 2026-01-11

**Authors:** Keimi Segami, Gabriela Garrido-Pinzás, Dyana Guardia, Fernando Mejía, Luis Malpica, Eduardo Gotuzzo

**Affiliations:** Instituto de Medicina Tropical “Alexander von Humboldt”, Lima, Lima, Peru; Instituto de Medicina Tropical “Alexander von Humboldt”, Lima, Lima, Peru; Instituto de Medicina Tropical “Alexander von Humboldt”, Lima, Lima, Peru; Instituto de Medicina Tropical “Alexander von Humboldt”, Lima, Lima, Peru; MD Anderson Cancer Center, Lima, Lima, Peru; Instituto de Medicina Tropical Alexander von Humboldt, Universidad Peruana Cayetano Heredia, Lima, Lima, Peru

## Abstract

**Background:**

Adult T-cell leukemia-lymphoma (ATL) is a peripheral T-cell malignancy caused by HTLV-1 infection. HTLV-1 carriers with a family history of ATL have a 12-fold increased risk of developing ATL. This study aimed to analyze ATL aggregation frequency in HTLV-1 carriers across generations.

**Figure:**
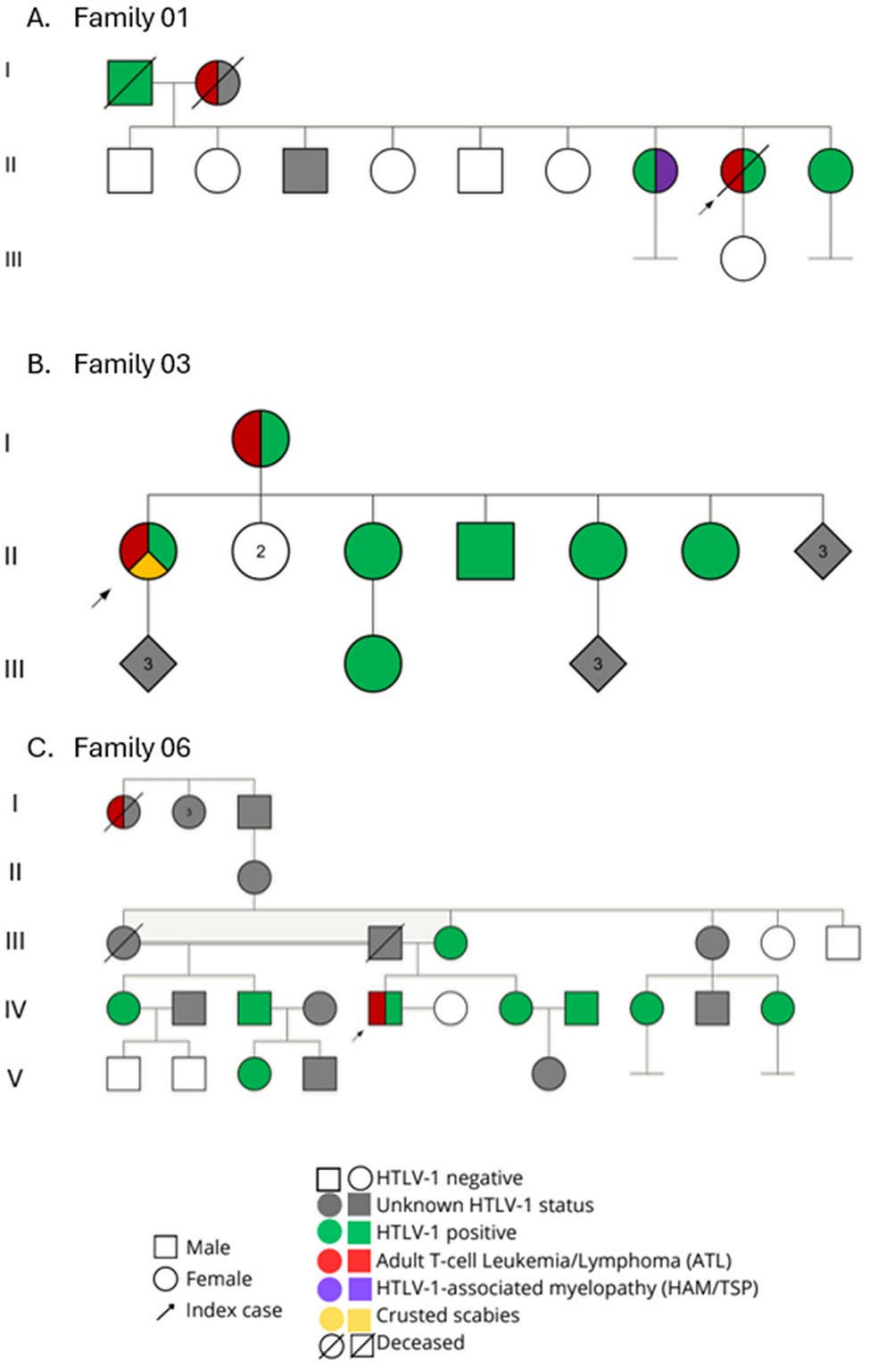
Genograms showing ATL aggregation in Families F1, F3, and F6 These families displayed complex multigenerational structures and were more extensively characterized. Each diagram illustrates vertical HTLV-1 transmission and multiple cases of Adult T-cell Leukemia/Lymphoma (ATL) within the same family.

**Figure:**
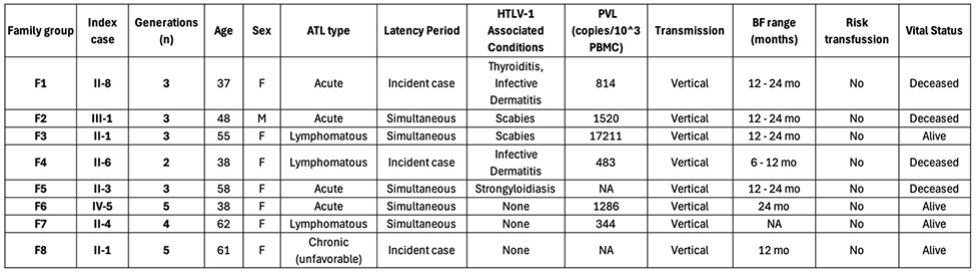
Overview of Clinical Features in Family Index Cases Demographic and clinical features of index cases from families with ATL aggregation, includes age, ATL subtype, latency period classification, HTLV-1–associated conditions, proviral load (PVL), transmission route, breastfeeding (BF) duration, risk transfusion history, and vital status. NA = Not Available.

**Methods:**

We performed a cross-sectional study between March and April 2025 at the HTLV-1 unit of the Instituto de Medicina Tropical Alexander von Humboldt (IMTAvH) in Lima, Peru, using data from a two-year investigation on HTLV-1-related malignancies conducted in 2023-2024, based on a clinical cohort established in 1992. We reviewed records of index cases with (1) confirmed HTLV-1 infection, (2) ATL diagnosis, and (3) a family history of ATL or leukemia/lymphoma. Clinical and epidemiological data from relatives were also reviewed. Only families with ATL aggregation (more than one case of ATL or related malignancy) were included. HTLV-1 seroprevalence was calculated as the number of seropositive individuals over the total number of relatives evaluated. ATL frequency was estimated using two ratios: (1) ATL cases among evaluated relatives, and (2) ATL cases among all identified family members. Descriptive statistics were reported as proportions for categorical variables and as means/medians for continuous variables. Genograms illustrate family relationships.

**Results:**

Twenty-six index cases led to the evaluation of their families. Eight families (30.8%) had multiple members with ATL and were included. HTLV-1 seroprevalence among family members was 55.17%. The median proportion of individuals with a diagnosis of ATL or lymphoma/leukemia per family was 22.65% (IQR: 17.5-25.0), representing 15.48% (IQR: 10.6-18.24) of individuals investigated. The median age was 51.5 years (IQR 38-59.5). Genogram analysis suggested vertical transmission as the primary route. Three index cases were incident ATL diagnoses, while others were diagnosed concurrently with HTLV-1 infection.

**Conclusion:**

Our findings highlight high intrafamilial seroprevalence of HTLV-1 and support ATL aggregation within families. This suggests that shared risk factors may contribute to disease clustering. Studying families with multiple ATL cases is essential for advancing our understanding and guiding prevention strategies.

**Disclosures:**

All Authors: No reported disclosures

